# Supplementary material for: The Neuron Phenotype Ontology: A FAIR Approach to Proposing and Classifying Neuronal Types
Source: Neuroinformatics. 2022 Mar 10;20(3):793–809. doi: 10.1007/s12021-022-09566-7 (PMC9547803; doi:10.1007/s12021-022-09566-7)
Supplement: Supplementary file 1 — Supplementary file1 (PDF 377 KB) [file 12021_2022_9566_MOESM1_ESM.pdf]

Supplemental Materials

Table S1: Criteria used to identify a CUT in the literature

1. The cell type is commonly referenced in introductory textbooks and teaching materials for neuroanatomy, cellular and systems neuroscience and neurophysiology.
2. The cell type is referenced in multiple articles that contain experimental findings from more than one modality and more than one laboratory or research project.
3. The cell type is generally recognized in multiple mammalian species.
4. The cell type is annotated and referenced in on-line knowledge bases, e.g., NeuroElectro.org[1], Hippocampome.org[2]
5. The cell type is referenced in on-line databases of experimental data, e.g., Blue Brain Project[3], NeuroMorpho[4].

Table S2: Abbreviations used in this paper. For transgenes and brain regions we provide links to additional information on their meaning where possible.

Abbreviation	Full name
Adcy1	Gene: adenylate cyclase 1
Adcy2	Gene: adenylate cyclase 2
Adcy8	Gene: adenylate cyclase 8
Adcy9	Gene: adenylate cyclase 9
Adm	Gene: adrenomedullin
Ai14(RCL-tdT)	Trangene: Ai14 is a Cre reporter allele designed to have a loxP-flanked STOP cassette preventing transcription of a CAG promoterdriven red fluorescent protein variant (tdTomato) - all inserted into the Gt(ROSA)26Sor locus. Ai14 mice express robust tdTomato fluorescence following Cre-mediated recombination. Used in Allen Cell Types Database
Ai65(RCFL-tdT)	Trangene: Target a Flp and Cre-dependent tdTomato expression cassette to the mouse Rosa26 locus used in Allen Cell Types Database (https://www.addgene.org/61577/)
Arhgef10	Gene: Rho guanine nucleotide exchange factor 10

[1] https://neuroelectro.org/
[2] http://hippocampome.org/php/index.php
[3] http://microcircuits.epfl.ch/#/article/article_3_mph
[4] http://neuromorpho.org/

BICCN	Brain Initiative Cell Census Network
Calb	Gene:calbindin 1
Calb2	Gene:calbindin 2 (calretinin)
Calca	Gene:calcitonin
CCK, Cck	Cholecystokinin
Cckbr	Gene:cholecystokinin B receptor
CCKC	Cholecystokinin small basket cells (Paul et al., 2017)
Chrm2	Gene:muscarinic acetylcholine receptor M2
Chrm3	Gene:cholinergic receptor, muscarinic 3, cardiac
CNP	Gene:C-type natriuretic peptide 3
Cnr1	Gene: protocadherin alpha 4
Cort	Gene:cortistatin
Cplx2	Gene: complexin 2
Cplx3	Gene: complexin-3
CR	Calretinin
Crh	Gene: corticotropin-releasing factor isoform 2 precursor
CQ	Competency query
CUT	Common usage type
DL	Description logic
EBT	Evidence based type[5]
Edn3	Gene: endothelin 3
Esrrg	Gene: estrogen-related receptor gamma
GABA	Gamma amino butyric acid
GABAR	GABA receptor
Gabra1	Gene: gamma-aminobutyric acid type A receptor subunit alpha1
Gabrd	Gene: gamma-aminobutyric acid (GABA) A receptor, subunit delta
Gria1	Gene: glutamate ionotropic receptor AMPA type subunit 1
Gria4	Gene: glutamate ionotropic receptor AMPA type subunit 4
GluR	Glutamate receptor

[5] In the data and documentation evidence based types are referred as evidence based models or EBMs which was their working name. As used here terms are equivalent.

Gpr88	Gene:G-protein coupled receptor 88
Grin3a	Gene:glutamate ionotropic receptor NMDA type subunit 3A
Gucy1a1	Gene:guanylate cyclase 1 soluble subunit alpha 1
Gucy1b1	Gene:guanylate cyclase 1 soluble subunit beta 1
Hcrtr1	Gene:hypocretin (orexin) receptor 1
Hs6st3	Gene: heparan-sulfate 6-O-sulfotransferase 3
Htr2c	Gene:5-hydroxytryptamine (serotonin) receptor 2C
Htr3a	Gene: 5-hydroxytryptamine receptor 3A
Igf1	Gene: insulin-like growth factor I
Kv3	Gene: potassium voltage-gated channel, shaker-related subfamily, member 3
Rspo2	Neuropeptide. This appears as Rspn in (Paul et al., 2017; Table S7) which we suspect is a typo.
Zip1	Gene:transcription factor Zip1
Znt3	Gene:zinc transporter 3
Inhbb	Gene:inhibin beta-B
LPC	Long projection cell of Paul et al. (2017)
Mef2c	Gene: myocyte-specific enhancer factor 2C
MNC	Martinotti cells (Paul et al., 2017)
Nefh	Gene: neurofilament, heavy polypeptide
NIF	Neuroscience Information Framework
NIFSTD	NIF Standard Ontology (Bug et al., 2008)
NOS	Gene:Nitric oxide synathase
NOS1-creER	Transgene: Also known as nNOS-CreER-KI knock-in allele, NOS1-creER was designed to both abolish neuronal nitric oxide synthase 1 (Nos1) gene function and direct CreERT2 fusion protein expression to nNOS positive GABAergic neurons by the endogenous Nos1 promoter/enhancer elements. (https://www.jax.org/strain/014541)
NOS1, Nos1	Nitric oxide synthase 1
NPO	Neuron Phenotype Ontology
NPOKB	Neuron Phenotype Ontology Knowledge Base, also the prefix on BioPortal.

npokb	Compact identifier prefix for NPO identifiers.
NPY, Npy	Neuropeptide Y
Npy1r	Gene: neuropeptide Y receptor Y1
Opn3	Gene: opsin 3
Oxtr	Gene: oxytocin receptor
Pde1a	calcium/calmodulin-dependent 3',5'-cyclic nucleotide phosphodiesterase 1A
Pde2a	Gene:phosphodiesterase 2A, cGMP-stimulated
Pde7b	Gene: cAMP-specific 3',5'-cyclic phosphodiesterase 7B
Penk	Gene: proenkephalin-A gene
Pnoc	Gene: prepronociceptin
Pparg	Gene: peroxisome proliferator-activated receptor gamma
Ppargc1a	Gene: PPARG coactivator 1 alpha
Prkg2	Gene: protein kinase, cGMP-dependent, type II
Prok2	Gene: prokineticin 2
Ptn	Gene: pleiotrophin
PV	Parvalbumin
PV-cre	Parvalbumin cre line
Pvalb-T2A-FlpO	Transgene: Also Known As:Pvalb-T2A-FlpO-D , Pvalb-2A-FlpO-D; Pvalb-T2A-FlpO-D (Pvalb-2A-FlpO-D) mice have both endogenous gene and optimized FLP recombinase (FlpO) gene expression directed to Pvalb-expressing cells by the endogenous promoter/enhancer elements of the parvalbumin (locus.https://www.jax.org/strain/022730)
PVBC	Parvalbumin basket cell (Paul et al. 2017)
Rasl11b	Gene: RAS-like family 11 member B
Rgs12	Gene: regulator of G-protein signaling 12
Rgs4	Gene: regulator of G-protein signaling 4
Rgs7	Gene:regulator of G-protein signaling 7
Rln1	Gene: prorelaxin H1
RSPd2/3	ABA Brain region: Retrosplenial area, dorsal part, layer 2/3 Structure in

	the Mouse Brain Atlas
S1	Primary somatosensory cortex
S7	Supplemental table 7 from Paul et al (2017)
Slc7a3	Gene: solute carrier family 7 (cationic amino acid transporter, y+ system), member 3
Slit2	Gene: slit guidance ligand 2
Slit3	Gene: slit guidance ligand 3
SPARC	Stimulating Peripheral Activity to Relieve Conditions
Sst-IRES-Cre	Transgene: somatostatin cre line developed by the Huang lab and used in Allen Cell Types Database
SST-flp, SST	Transgene: somatostatin-ires-Flpo (Huang neurons). Same as Sst-IRES-FlpO
Sst-IRES-FlpO	Transgene: FLPo recombinase expression in GABAergic neurons derived from the medial-ganglionic eminence (MGE) - as directed by the endogenous somatostatin promoter developed by Huang lab and used in the Allen Cell Types Database
Sst, SS	Somatostatin
Syt10	Gene: synaptotagmin 10
Syt4	Gene: synaptotagmin 4
Syt5	Gene: synaptotagmin 5 (synaptotagmin 9)
Syt6	Gene: synaptotagmin 6
Tac1	Gene: tachykinin precursor 1
Tac2	Gene: tachykinin 2
Tacr1	Gene: tachykinin receptor 1
Trpc6	Gene: transient receptor potential cation channel subfamily C member 6
Unc5d	Gene: unc-5 netrin receptor D
VIP	Vasoactive intestinal polypeptide
Vipr1	Gene: vasoactive intestinal peptide receptor 1
Vipr2-IRES2-Cre	Transgene: Vipr2-IRES2-Cre-D knock-in mice (https://www.jax.org/strain/031332) In subcortical areas, this Cre line has expression in arcuate hypothalamic nucleus, dorsal lateral geniculate nucleus, ventral nucleus of the thalamus, bed nuclei of the

	stria terminalis, and scattered expression in superior colliculus. In the cortex, there is scattered expression in neurons and non-neuronal cells. (from Allen Cell Types Database documentation)
VISl5	ABA Brain Region: Lateral visual area, layer 5
VISp6a	ABA Brain Region: Primary visual area, layer 6a
VISrl2/3	Allen Brain Atlas:Rostrolateral area, layer 2/3
Wnt2	Gene: Wnt-2 protein

Table S3: Comparison with other Cell Ontologies in Bioportal

Ontology	Description	Ontology features	NPOKB features
Cell Ontology[6]	The Cell Ontology (CL) is designed as a structured controlled vocabulary for cell types. This ontology was constructed for use by the model organism and other bioinformatics databases, where there is a need for a controlled vocabulary of cell types. This ontology is not organism specific it covers cell types from prokaryotes to mammals. However, it excludes plant cell types, which are covered by PO. See the website (From BioPortal[7])	• Covers all cell types<br>• Built on BFO; all neurons are direct children of electrically active cell<br>• Makes use of OWL axioms<br>• Anatomical location assigned to entire cell using the "Part of" relationship<br>• Imports UBERON, CHEBI, Protein Ontology, GO<br>• Makes extensive use of GO biological processes to related molecules to cell, e.g., Neurotransmitter captured through GO biological process "neuron and ('capable of' some 'acetylcholine secretion, neurotransmission')"<br>• NPOKB CUTs should be imported into CL	• Focused on neurons<br>• Built on BFO; Neuron CUTs and EBTs are explicit classes under Neuron<br>• Makes use of OWL axioms<br>• Anatomical location assigned by location of soma using the "HasSomaLocatedIn" property<br>• Uses UBERON for anatomical locations<br>• Uses more direct relationships to relate molecules to cell, e.g., Neurotransmitter assigned by property "haNeurotransmitter"
Provisional Cell Ontology	Provisional cell ontology (PCL) is based on results of transcriptomic and developmental studies of brain largely carried out at the Allen Institute for Brain Science (Hodge et al. 2019)	• All cells are children of OWL: Thing; not asserted to be cells<br>• Captures developmental information<br>• No OWL axioms<br>• Does not model molecular phenotypes<br>• Uses UBERON for anatomical locations	• All neurons are children of "Neurons"> Nervous system cell > cell<br>• Makes use of OWL axioms<br>• Uses UBERON for anatomical locations + brain atlas regions<br>• Molecular phenotypes

[6] http://cellontology.org
[7] https://bioportal.bioontology.org/ontologies/CL

			modeled through OWL axioms<br>• Does not capture developmental phenotypes
NeuroMorpho Brain Region Ontologies [8]	The species, brain regions and cell types are integrated into a single ontology for enabling OntoSearch functionality to mine the latest v7.0 release of NeuroMorpho.Org. (from BioPortal)	• All cells are children of OWL:thing<br>• No OWL axioms<br>• Minimum number of properties<br>• Does not model molecular or location phenotypes<br>• Has invertebrate neurons	• All neurons are children of "Neurons"> Nervous system cell > cell<br>• Makes use of OWL axioms<br>• Uses UBERON for anatomical locations + brain atlas regions<br>• Molecular phenotypes modeled through OWL axioms<br>• No invertebrate neurons

Table S3 legend: Cell ontologies similar to NPO in Bioportal. Ontology = name of ontology; Description = description of ontology; Ontology features= Some features from the other ontologies; NPOKB features = corresponding features of NPO to highlight similarities and differences. Of the three, NPO is closely aligned with the Cell Ontology (Diehl et al. 2016) *in design in that they are built from the BFO upper ontology and import many of the same community ontologies. They do make different modeling decisions, however, on key phenotypes, so they are not entirely interoperable. It is also similar in scope to the Provisional Cell Ontology* (Hodge et al. 2019) *in that it focuses on cell types derived from experimental techniques. However, the former does not make as extensive use of community ontologies, and very little use of the logical features of OWL to aid in classification. BFO = basic formal ontology*[9]

Figure S4: Interconversion between representations of neuron types enabled by neurondm. Conversion to and from tabular formats such as google sheets, and OWL/RDF serializations is implemented for the core Neuron Lang equivalentClass of intersection of owl restrictions

[8] https://bioportal.bioontology.org/ontologies/NMOBR/?p=summary

[9] https://basic-formal-ontology.org/#:~:text=The%20Basic%20Formal%20Ontology%20(BFO,is%20a%20genuine%20upper%20ontology.&text=BFO%20is%20used%20by%20more,driven%20endeavors%20throughout%20the%20world.

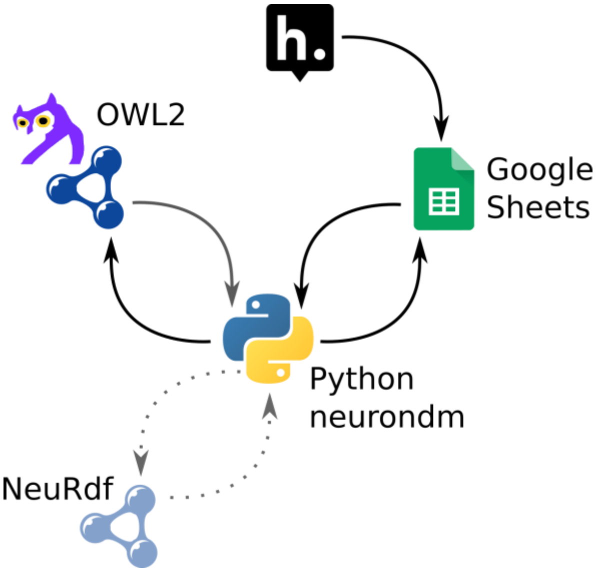

pattern. Tabular formats that support notes on cells can include links to hypothes.is[10] annotations that point to sections of papers that provide evidence to support the presence of a particular neuron phenotype. Annotations can be represented in RDF, but are only easily accessible in a blank-node free representation such as NeuRDF. The NeuRdf format has not been fully implemented.

Supplemental methods:

Working with the NPO

The easiest way to work with the NPO is to use the docker images that have been correctly configured for working with the NIF-Ontology, Protégé, and neurondm. Additional details are provided below for users who want to configure their own system to work with the NPO.

The use the docker image for the NPO 1.0 run the following commands after installing docker and ensuring that docker images can connect to an X11 server. The npo-1.0 docker image is configured to load all content locally from the NIF-Ontology repository so external ontologies are not pulled in and the image is stable. In addition to docker you will need an X11 server installed and running your operating system, for macos we have tested with xquartz, and on windows with vcxsrv.

If you are on linux you can run the following.

```
xhost local:docker
docker pull tgbugs/musl:npo-1.0-neurondm-build
docker run \
```

[10] https://hypothes.is

```
-v /tmp/.X11-unix:/tmp/.X11-unix \
-e DISPLAY=$DISPLAY \
--workdir /home/protegeuser/git/NIF-Ontology/ttl \
tgbugs/musl:npo-1.0-neurondm-build \
sh -c 'git stash && protege ~/git/NIF-Ontology/ttl/npo.ttl'
```

If you are on macos you can run the following.

```
xhost localhost
docker pull tgbugs/musl:npo-1.0-neurondm-build
docker run \
-v /tmp/.X11-unix:/tmp/.X11-unix \
-e DISPLAY=host.docker.internal:0 \
--workdir /home/protegeuser/git/NIF-Ontology/ttl \
tgbugs/musl:npo-1.0-neurondm-build \
sh -c 'git stash && protege ~/git/NIF-Ontology/ttl/npo.ttl'
```

If you are on windows you can run the following.

```
docker pull tgbugs/musl:npo-1.0-neurondm-build
docker run \
-e DISPLAY=host.docker.internal:0 \
--workdir /home/protegeuser/git/NIF-Ontology/ttl \
tgbugs/musl:npo-1.0-neurondm-build \
sh -c 'git stash && protege ~/git/NIF-Ontology/ttl/npo.ttl'
```

If you run this on macos you will need to increase the memory limit to 8gigs to avoid protege from being killed with an out of memory error when it uses more than 2gis of memory. This can be done in Docker Desktop on macos in Preferences > Resources > Advanced. In addition on macos you should run xhost +localhost instead of xhost local:docker. Finally, the DISPLAY environment variable should be set to host.docker.internal:0 on macos and host.docker.internal:0.0 on windows. For the most recent instructions see the latest version of the docker file sources[11].

The neurondm library depends on a set of core ontology files that define the predicates and the different types of neuronal phenotypes that can be used to describe a neuron type. These files are packaged as part of neurondm releases. It is possible to use most of the neurondm tooling without a local copy of the NIF-Ontology repository. For development neurondm requires a local copy of the NIF-Ontology repository with the neurons branch[12] checked out.

[11] https://github.com/tgbugs/dockerfiles/blob/master/source.org
[12] https://github.com/SciCrunch/NIF-Ontology/blob/master/docs/Neurons.md

Detailed documentation on how to load the NPO into the Protégé Ontology Tool[13] is maintained in GitHub.[14] However, the NIFSTD is a very large ontology with multiple import statements that can be difficult to work with. First, loading the full ontology in Protégé requires retrieving all files in the import chain and patching the ontology catalog file and any external ontologies with known issues. Once this is done, it is possible to load the ontology and run the reasoner. The full NIFSTD ontology is relatively large and reasoning over it and the full set of predicates available for describing phenotypes requires roughly 4 gigs of memory at the outset which grows as the reasoning proceeds. On a quad-core machine from 2013 loading takes about 30 seconds and initial reasoning about another 10 seconds, however incremental reasoning thereafter can take many minutes. In practice we have found that it can sometimes be more expedient to close Protégé, reload NIFSTD, and reason from scratch than to use incremental reasoning when there are changes. The fully expanded and reasoned NIF ontology is also accessible via a SciGraph API endpoint (see using NIFSTD for details) that serves a graph built using an automated version of the loading process. SciGraph is a Neo4j backed ontology store that is used by the python representation of the NPO to obtain lexical information about phenotypes and to verify that they are in the set of known terms if the term is not part of the core classes that are stored locally.

To make the NPO more accessible for inspection and use by subject matter experts, we created NPO "Light", a stripped down .ttl version that loads in most desktop installations of the Protégé Ontology Browser (version 5.5 or higher)[15] without the requirement to download the full NIFSTD or any additional modifications to the local machine. NPO Light can be loaded into Protégé on either a PC or Mac using the "Open from URL" option in the file menu. NPO Light supports basic reasoning about neuron types, but does not support the full reasoning chain. In addition, the NPO Light file includes the genes, proteins and brain regions that are used in the current set of neuron phenotype definitions, i.e., NPO Light does not import the full gene list of NCBI gene. Unless otherwise specified, all of the examples presented in this work were derived from NPO Light.

Structure of the NPO

The NIF Ontology makes extensive use of OWL import statements to combine a variety of ontology source files and community ontologies into a single whole. Within the NIF Ontology as defined by nif.ttl, the content of the NPO is imported via a bridge file[16]. This file imports the core phenotype ontology files2[17] as well as the file that defines the common usage types and the files that define the evidence based types[18]. The core phenotype ontology files include the OWL properties for phenotypic dimensions, and phenotypes that are specific to neurons. The NPO light ontology that is defined by npo.ttl also works by importing neuron-bridge.ttl, however the

13 https://protege.stanford.edu/
14 https://github.com/SciCrunch/NIF-Ontology/blob/master/docs/Neurons.md#protege-development-setup
15 https://raw.githubusercontent.com/SciCrunch/NIF-Ontology/npo-1.0/ttl/npo.ttl
16 https://github.com/SciCrunch/NIF-Ontology/blob/npo-1.0/ttl/bridge/neuron-bridge.ttl
17 https://github.com/SciCrunch/NIF-Ontology/blob/npo-1.0/ttl/phenotype-core.ttl
https://github.com/SciCrunch/NIF-Ontology/blob/npo-1.0/ttl/phenotypes.ttl
18 https://github.com/SciCrunch/NIF-Ontology/tree/npo-1.0/ttl/generated/neurons

rest of the imports that are present in the NIF Ontology are excluded. Instead, we extract only the subset of the terms from the ontology that are used as phenotype values along with parent terms in the subClassOf and part of hierarchies and the subset of triples that are needed to be able to get the same results when running our competency queries as in the full ontology. We also include triples needed to make the light ontology human readable when loaded in Protégé so that e.g. the terms appear with their rdfs:label instead of as curies.

The NPO as a whole can thus be considered to be a combination of the core phenotype ontology files and the NPOKB which includes the CUTs and the EBTs.

Modeling Location Phenotypes

For location based queries the objective is to be able to find neurons that are located in some region. Human beings automatically fill in the implied "or any part of that region," however, the ontology cannot do this automatically. To accomplish this we do two things. First we lift phenotypes of the that are defined using location predicates of the form =?subject hasLocationPhenotype: :Location= into the form =(hasLocationPhenotype: SOME (partOf: SOME :Location))=. Second we add a partOfSelf axiom =:Location SubClassOf: (partOf: SOME :Location)= to all anatomical entities that are used as phenotype values. This solution provides query behavior that matches what human beings expect when they write a DL query of the form =hasPhenotype some ('part of' some Location)=. This structure is also compatible with both the FaCT++ and ELK reasoners. A query of the form =hasSomaLocatedIn some Location= does work if a subPropertyChain axiom of the form =hasLocationPhenotype: owl:propertyChainAxiom (hasLocationPhenotype: partOf:)= is added to the ontology, however this only works with the ELK reasoner since inclusion of that axiom crashes FaCT++. For more details on the modelling of location phenotypes please see the documentation on OWL modelling decisions [19].

Atlas regions vs anatomical names

A continuing challenge in building ontologies for neuroscience has been the large number of anatomical terminologies derived from brain atlases and parcellation schemes that must be reconciled when referring to anatomical locations. The anatomical locations for CUTs are expressed in generic anatomical terms using the pan-species UBERON ontology (Mungall et al. 2012). Because EBTs and instances are tied directly to experimental data, locations may be expressed in terms of a particular brain atlas rather than a generic anatomical term, e.g., the Paxinos and Franklin mouse brain atlas (Franklin and Paxinos 2008) or the Allen Mouse Brain Reference Atlas (RRID:SCR_013286) terminology.

For the NPO, we enhanced NIF's core anatomical ontologies by creating a parcellation scheme ontology[20]. The current version contains ~ 30 nomenclatures from major brain atlases in rat, mouse, human and cat. For each parcellation scheme, we either imported the hierarchy if it was available, e.g., Allen Brain Mouse Atlas, or created a hierarchy based on region names and information provided with the nomenclature. This process sometimes necessitated creating additional superclasses that were not present in the original nomenclature. For example, the Paxinos atlases divides the cerebellum up into subregions, but does not contain the root:cerebellum. To relate atlas structures to common brain regions, we utilize the "delineated by" relationship, e.g.,

[19] https://github.com/tgbugs/pyontutils/blob/master/neurondm/docs/basic-model.org

[20] http://ontology.neuinfo.org/NIF/ttl/generated/parcellation-artifacts.ttl

Neocortex +SOM neuron = neuron and hasPhenotype some ('part of' some (neocortex or (delineates some neocortex))) and hasPhenotype some (Sst or SST or Sst-IRES-Cre or Sst-IRES-FlpO)

This DL query returns neurons that have their soma located in any part of the neocortex or in any atlas structure that delineates a subregion of the neocortex.

Converting from opaque sources

The opaque and computationally inaccessible nature of some of the original sources for the knowledge that we are encoding is a significant obstacle to validating the correctness of the ontological models that we have created both for ourselves and for others.

This is not just an obstacle for this paper, but for anyone trying to work with the original publications. The opaqueness of the knowledge representation is a major negative externality that is a drain on the productivity and resources of every single lab that wants to reuse or validate this knowledge.

Worse still, every time such opaque knowledge is translated into some computationally tractable form, new and different mistakes and errors creep in. We have worked repeatedly to detect and correct errors in the original sources and errors that we ourselves introduced during conversion.

In all cases there is no mechanical means for validating that our conversions are correct beyond having someone with enough motivation manually review both the original source and the derivative product. In the best case we would be able to regenerate the original figures in a reproducible manner from a computationally accessible representation, but at a certain point we have to ask whether this is our job as ontologists and curators or whether this is something that should be expected as a matter of course from the original authors.
